# Safety and efficacy of dehydrated ethanol soaking of the operative field in the treatment of spontaneous hepatocellular carcinoma rupture

**DOI:** 10.1186/s12957-018-1390-x

**Published:** 2018-04-26

**Authors:** Jian Sun, Yue Zhu, Yao-rong Peng, Wen-bin Li, He-yun Zhang, Zhen-yu Zhou, Lin Wang, An-de Ma, Jie Wang

**Affiliations:** 1 Guangdong Provincial Key Laboratory of Malignant Tumor Epigenetics and Gene Regulation, Medical Research Center, Sun Yat-Sen Memorial Hospital, Sun Yat-Sen University, Guangzhou, 510120 China; 2 Department of Hepatobiliary and pancreatic Surgery, Sun Yat-Sen Memorial Hospital, Sun Yat-Sen University, Guangzhou, 510120 China; 3 Department of Vascular and Thyroid Surgery, Sun Yat-Sen Memorial Hospital, Sun Yat-Sen University, Guangzhou, 510120 China; 4 Department of Pathology, Sun Yat-Sen Memorial Hospital, Sun Yat-Sen University, Guangzhou, 510120 China; 5 Center for Hygiene Testing and Analysis, Southern Medical University, Guangzhou, 510120 China

**Keywords:** Dehydrated ethanol, Spontaneously rupture, Hepatocellular carcinoma

## Abstract

**Background:**

The aim of our study was to evaluate the clinical safety and value of ethanol surgical field infiltration (ESFI), combined with distilled water peritoneal lavage (DWPL), after hepatectomy in patients with hepatocellular carcinoma (HCC) rupture.

**Methods:**

Rat liver tissue samples were soaked in dehydrated ethanol for different soaking times, and 18 rats were assigned to three groups that underwent different soaking methods of the hepatectomy cut surface. We retrospectively reviewed 45 patients who underwent hepatectomy for treatment of ruptured HCC. Among these, EFSI combined with DWPL was used in 21 patients (DAW group), with only DWPL used in the other 24 patients (DW group). Clinical outcomes were compared between the two groups.

**Results:**

For in vitro experiments, the depth of coagulation degeneration and necrosis increased with the duration of soaking. For in vivo experiments, rats in all three groups survived until postoperative day 7 without significant postoperative complication. In patients, the rate of post-operation complication was comparable between the two groups (*P* = 0.398), with no between-group differences in liver function levels. The incidence of peritoneal dissemination was significantly higher for DW than DAW group (*P* = 0.037). Kaplan–Meier test identified dehydrated ethanol treatment as a significant factor of disease-free survival (DFS) (*P* = 0.036). On univariate analysis, dehydrated ethanol treatment was associated with better prognostic outcomes, although it was not retained as an independent factor of patient outcome.

**Conclusions:**

Dehydrated ethanol soaking of the cut surface of the hepatectomy could potentially lower the risk of metastasis and improve the effect of hepatectomy for ruptured HCC as well as showed potential therapeutic value for intraoperative iatrogenic rupture of HCC.

**Electronic supplementary material:**

The online version of this article (10.1186/s12957-018-1390-x) contains supplementary material, which is available to authorized users.

## Background

Hepatocellular carcinoma (HCC) is the sixth most prevalent cancer, with an age-adjusted worldwide incidence of 16 cases per 100,000 individuals [1, 2]. Spontaneous rupture of HCC is a life-threatening situation that is associated with a poor prognosis, even when treated with surgical resection [3, 4]. According to the TNM staging system of the AJCC/UICC (American Joint Committee on Cancer/Union for International Cancer Control), all ruptured HCC tumors are assigned a grade of T4 [5]. Although some studies argue that it is an overestimation to assign a T4 classification to all resectable ruptured tumors, others have demonstrated that spontaneous HCC rupture carries a specific additional negative impact on overall survival (OS) and disease-free survival (DFS) and, therefore, that a 0.5 to 2 stage classification should be added to the baseline tumor stage [6, 7].

Curative liver resection is the most effective treatment for ruptured HCC to improve patient survival. However, this treatment is associated with a high rate of tumor recurrence due to the risk of implanted metastases [6, 8]. Several methods have been developed in an attempt to decrease the incidence of metastases, including peritoneal lavage with distilled water (DWPL). This conventional technique has been widely used to remove bacteria and tumor cells from the abdominal cavity. However, metastatic tumor cells tend to be agglomerated, rather than existing as individual cells, which makes them difficult to simply be eliminated. Moreover, the cytocidal activity of the DWPL technique is decreased by contamination of the water once it is in contact with the peritoneal cavity, as well as by the limited time window available for lavage to be successful [9, 10].

Dehydrated ethanol injected percutaneously (PEIT) has been used for the treatment of nodular-type HCC due to its accessibility and cost-efficacy [11]. In the case of ruptured HCC, PEIT by laparotomy or under ultrasound guidance has been successfully used to stop the bleeding [12, 13]. Based on this evidence, we hypothesized that dehydrated ethanol could be applied directly on the residual cavity of hepatectomy for ruptured HCC to improve clinical outcomes. Therefore, the aim of our study was to evaluate the safety of using dehydrated ethanol in the operating field in an animal model, as well as to investigate the clinical safety and value of combining ethanol surgical field infiltration (ESFI) with DWPL during hepatectomy performed in patients for the treatment of ruptured HCC.

## Methods

### Animals

Twenty male Wistar albino rats, with a body weight of 200–250 g, were obtained from the Central Animal House of the Sun-Yat Sen University (Guangzhou City, PR China). All animal procedures were conducted according to the guidelines of our institutional animal care center for the ethical use of animals in research, and the protocol was reviewed and cleared by our institution’s research ethics committee.

### In vitro experiment and histopathological analyses

Laparotomy was performed in two rats to harvest the left lobe of the liver. The resected liver tissue was cut into 1-cm slices, with tissue slices randomly assigned to the following duration of soaking in a dehydrated ethanol solution: 30 s and for 1, 3, 5, 10, and 30 min. Slices soaked in saline were used as a control. All liver tissues underwent routine histopathological examination including hematoxylin-eosin (H&E) and periodic acid-Schiff (PAS) staining. The depth of coagulation necrosis and the extent of fibrous degeneration and infiltration of inflammatory cells were examined in each slice. As well, vessels and bile duct were identified in each slice.

### In vivo experiment

**Fig. 1:**
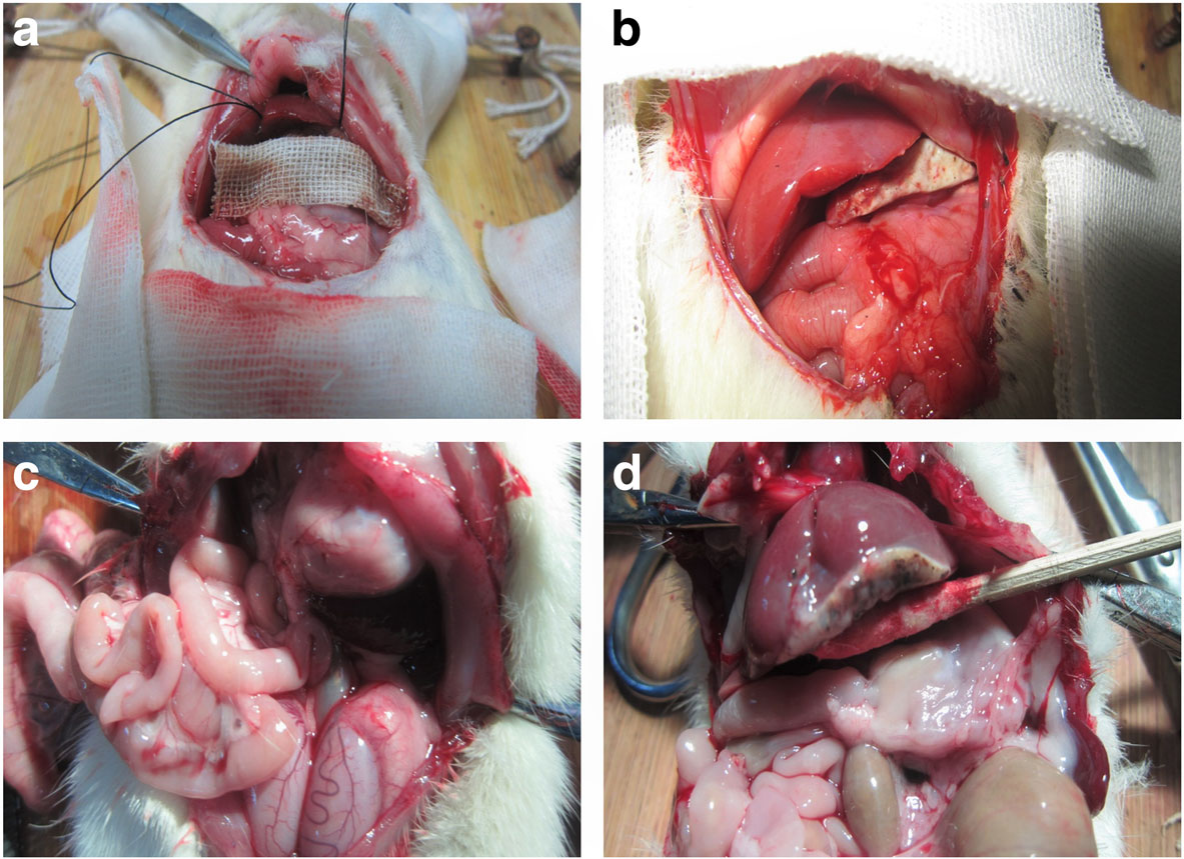
In vivo experiment. **a** Partial hepatectomy was performed through a 2.5-cm subcostal incision for 3 groups of rats that underwent different conditions of hydropathic compression of the cut hepatectomy surface. At the time of sacrifice, no severe abdominal adhesions were identified in DW group (**b**), DAW-3-min group (**c**), and DAW-5-min group (**d**)

Partial hepatectomy was performed in 18 rats. Serum specimens were collected from the caudal vein preoperatively. To evaluate the effects of ethanol of tissue, animals were randomly assigned to three groups undergoing different conditions of hydropathic compression of the cut hepatectomy surface. In group A, the surface was compressed with an ethanol gauze for 3 min; in group B, with an ethanol gauze for 5 min; and in group C, with a saline gauze for 5 min, serving as the control. The ethanol and saline solutions were continuously dripped onto the gauze for the specified duration (Fig. 1a). All rats were sacrificed on postoperative day 7, and 3-ml intracardiac blood samples were drawn followed by examination for intraperitoneal adhesions of the bowel. AST (aspartate transaminase), ALT (alanine transaminase), ALB (albumin), and TB (total bilirubin) levels were measured from the venous and intracardiac blood samples.

### Retrospective case study

A retrospective cohort study was performed to analyze 45 patients who were treated at our hospital for a ruptured HCC between March 2005 and March 2013: 37 males and 8 females, 44.9 ± 11.7 years (range, 22–81 years), with no history of allergy to alcohol. All patients underwent liver resection, with alcohol soaking performed in the residual cavity of the operative field, followed by DWPL, in 21 patients (DAW group), with the other 24 patients treated only with DWPL as a control group (DW group). Relevant demographic, medical history, and clinical variables were recorded. The severity of liver disease was classified according to the Child-Pugh criteria. Vascular invasion was determined by microscopic and macroscopic infiltration on histopathological examination of tissue samples. Our study protocol involving patients was approved by the Institutional Review Board of Sun Yat-Sen University (Guangzhou, P. R. China), and all patients provided informed consent.

**Fig. 2:**
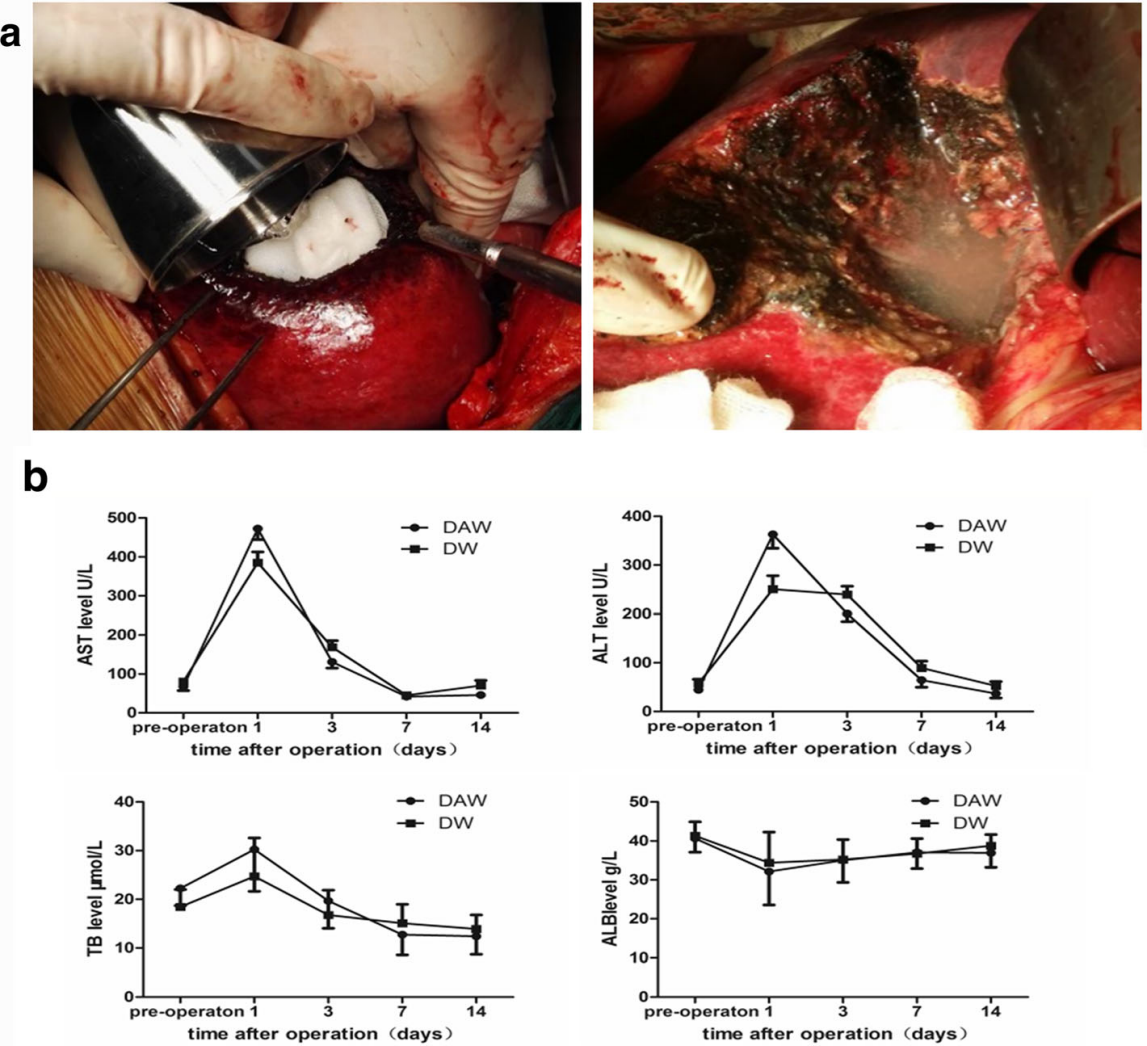
Retrospective case study. **a** The cut surface of the hepatectomy was soaked with 50–100 ml of dehydrated alcohol for 5 min; then, the residual anhydrous alcohol films were cleaned by repeated 5-min saline wash until no deposits were visible. **b** Levels of AST, ALT, TB, and ALB between the groups at all time points of measurement

For dehydrated alcohol soaking, the residual cavity of the operative field was surrounded with saline gauze and the cut surface of the hepatectomy was soaked with 50–100 ml of dehydrated alcohol for 5 min. With this process, visible floc becomes visible around the wound and in the surrounding liquid. The anhydrous alcohol was then suctioned off, and the residual anhydrous alcohol film cleaned by a repeated 5-min saline wash until no deposits were visible (Fig. 2a).

For distilled water peritoneal lavage, the residual cavity was washed with 5–6 L of distilled water, divided into at least 3 cycles. In each cycle, the peritoneal cavity was filled with warm (35–40 °C) distilled water and retained for 3 min. The water was then suctioned from the abdominal cavity. The process was repeated until the entire volume of distilled water had been applied, with the entire procedure taking, on average, 15 min.

### Statistical analyses

All data were analyzed using the Statistical Package for Social Sciences (SPSS; version 16.0 for Windows; SPSS Inc., Chicago, IL). Between-group differences were evaluated using Kruskal-Wallis or Mann-Whitney *U* tests, depending on the normality of the underlying distribution. Data were expressed as a mean ± SD or median (interquartile range), as appropriate. The significance of clinicopathological features in the 45 patients with ruptured HCC carcinoma was evaluated using chi-squared analysis. Survival rates were calculated using the Kaplan–Meier method, with overall OS defined as the time from hepatectomy until death from any cause or the end of the observation period. DFS was defined at the time from hepatectomy until recurrent disease detection or the end of the observation period without recurrence. Univariate analysis was performed to identify prognostic factors of OS and DFS, with significant variables retained for multivariate analysis and logistic regression. A *P* value < 0.05 was deemed to be significant.

## Results

### In vitro experiment

**Fig. 3:**
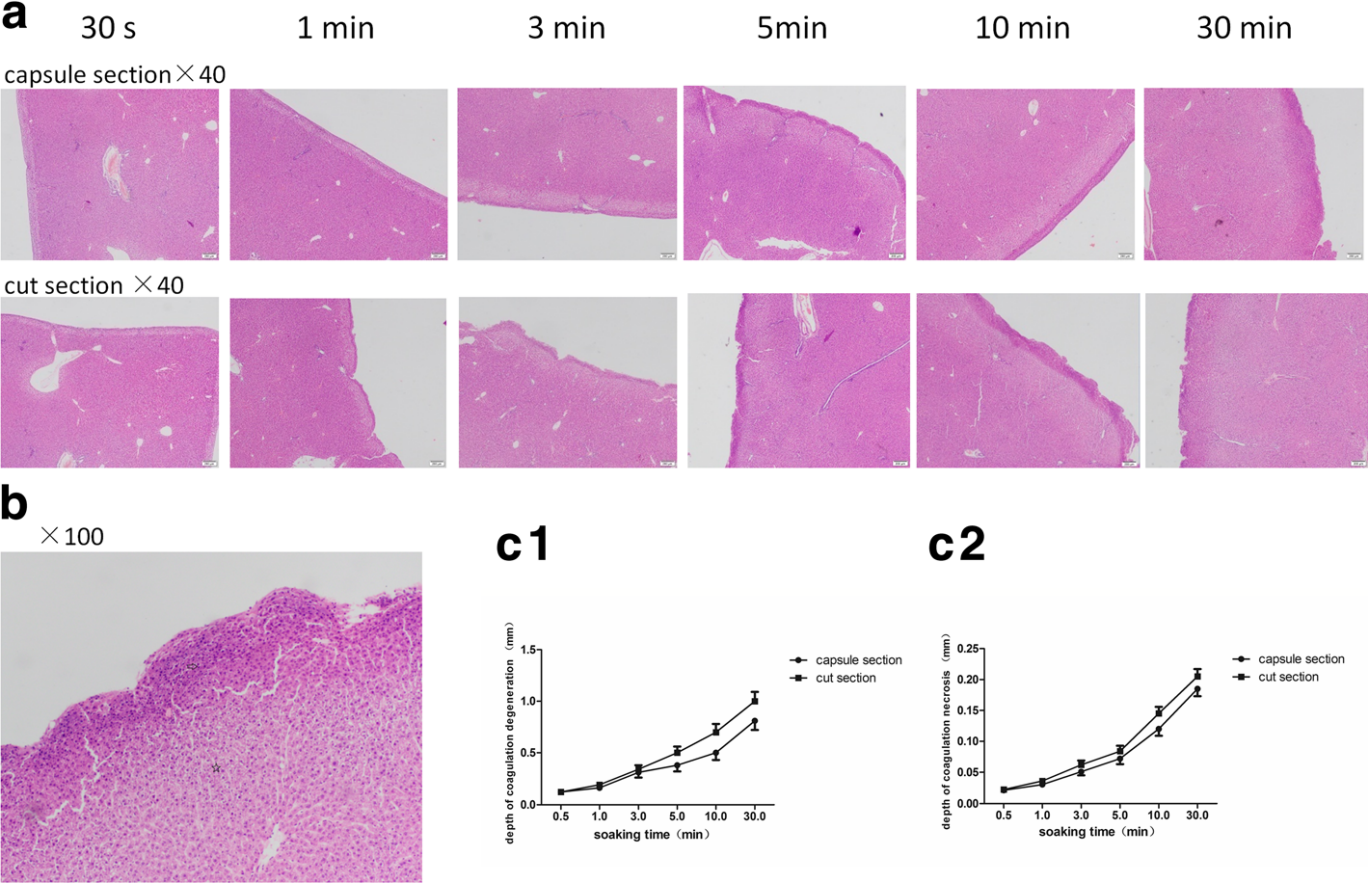
In vitro experiment. **a** HE staining of liver tissues for the depth of coagulation necrosis and coagulation degeneration at different soaking times in dehydrated ethanol. × 40 magnification. **b** After being soaked in the alcohol, the liver cells emerged to shrivel. Several layer cells of the surface appeared spindle; their cytoplasm was decreased and stained deep (arrow). Some cells appeared to be necrosis, and nuclear condensation or apoptotic bodies in the apoptotic cells were observed with time last. Below the surface layer cells, there were several layers of degeneration cells. These cells had plenty of plasma as the swelling of cells. Therefore, these cells appeared more larger and paler than normal liver cells (star). × 100 magnification. **c** The depth of coagulation necrosis and degeneration increased as a function of the duration of soaking in dehydrated ethanol, for both tissue samples obtained from the cut hepatectomy surface and the capsule section of the liver

On histological examination, hepatocytes shriveled after soaking in the dehydrated ethanol. Several layers of cells on the surface of tissue samples were spindle-shaped, with decreased cytoplasm that was deeply stained. Nuclear condensation and apoptotic bodies were identified within hepatocytes, with an area of necrosis observable on the most lateral surface of the liver tissue samples. Tissue degeneration, with swelling and pale cells, was identified between the area of necrosis and the inner layers of the tissue samples consisting of normal hepatocytes (Fig. 3b). The depth of coagulation necrosis and degeneration increased as a function of the duration of soaking in dehydrated ethanol, for both tissue samples obtained from the cut hepatectomy surface and the capsule section of the liver (Fig. 3a, c; Table 1).

**Table 1:** The depth of coagulation necrosis and degeneration with the duration of dehydrated ethanol soaking

	Cut hepatectomy surface		Capsule section	
Time	Coagulation necrosis	Coagulation degeneration	Coagulation necrosis	Coagulation degeneration
30 s	0.022 ± 0.002	0.12 ± 0.010	0.021 ± 0.002	0.12 ± 0.010
1 min	0.036 ± 0.004	0.19 ± 0.020	0.033 ± 0.003	0.16 ± 0.020
3 min	0.062 ± 0.007	0.34 ± 0.040	0.051 ± 0.006	0.31 ± 0.050
5 min	0.084 ± 0.009	0.50 ± 0.060	0.072 ± 0.009	0.38 ± 0.060
10 min	0.145 ± 0.010	0.70 ± 0.080	0.120 ± 0.011	0.50 ± 0.070
30 min	0.205 ± 0.012	1.00 ± 0.090	0.185 ± 0.012	0.81 ± 0.090

Data were expressed as a mean ± SD (mm)

### In vivo experiment

**Fig. 4:**
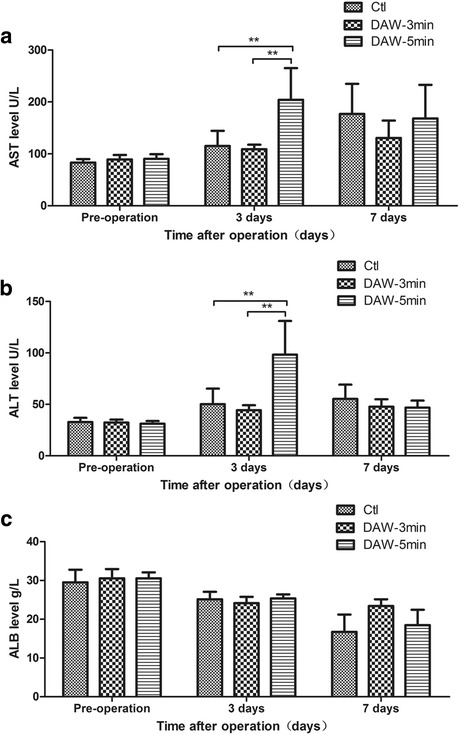
The liver function examination on postoperative days 3 and 7 of rats including AST(**a**), ALT(**b**) and ALB(**c**)

All rats survived until postoperative day 7 without significant postoperative complications. At the time of sacrifice, no severe abdominal adhesions were identified in any group (Fig. 1b–d). On liver function examination, AST level was significantly higher in group B (5-min ethanol soak) than that in groups A (3-min ethanol soak; *P* = 0.028) or C (control; *P* = 0.017) on postoperative day 3. ALT level was similarly higher in group B than in groups A (*P* = 0.009) or C (*P* = 0.014) on postoperative day 3. Levels of AST and ALT were comparable between three groups on postoperative day 7. ALB level was also comparable between the three groups on both postoperative days 3 and 7 (Fig. 4).

### Retrospective case study

The clinicopathological features of the 45 patients who underwent curative hepatectomy for a ruptured HCC are summarized in Table 2, with no significant differences between the DAW and DW groups, and all patients surviving the perioperative period. Among the 21 patients in the DAW group, 6 patients developed postoperative complications, including 4 patients with a hydrothorax, 1 with ascites and 1 with a peritoneal infection. Among the 24 patients in the DW group, 5 developed postoperative complications, including 2 patients with a hydrothorax, 2 with ascites, and 1 with a pulmonary embolism. Overall, there was no difference in the incidence rate of postoperative complications between the DAW and DW groups (Table 3; *P* = 0.398). Levels of AST, ALT, ALB, and TB were comparable between the groups at all time points of measurement: preoperatively and on postoperative days 1, 3, 7, and 14 (Fig. 2b). Ethanol concentration was evaluated in six patients who underwent hepatectomy followed by ethanol soaking to confirm safety of the dehydrated ethanol procedure after hepatectomy. The maximum blood ethanol concentration identified was 30.1 mg/dL, which is safe for humans (Additional file 1: Table S1).

**Table 2:** Demographic and clinical characteristics of all patients included in the study

	DAW group	DW group	*P* value
	*N* = 21	*N* = 24	
Age, years	48.6 (22-81)	46.8 (22-71)	0.688
Male to female	17:4	20:4	1.000
HBV positivity^a^	16 (76.2%)	21 (87.5%)	0.549
Tumor location			0.607
Left lobe	5 (23.8%)	6 (25%)	
Right lobe	12 (57.1%	15 (62.5%)	
Both left and right lobe	4 (19.1%)	3 (12.5%)	
Child-Pugh classification^a^			0.551
A	20 (95.2%)	22 (91.7%)	
B	1 (4.8%)	2 (8.3%)	
C	0	0	
Pre-operation TACE^a^	0	3 (12.5%)	0.236
AST, IU/L	70.7 (29–160)	78.6 (22–347)	0.795
ALT, IU/L	47.8 (16–162)	59.3 (26–152)	0.507
AFP, IU/mL	45,705.0 (1.14–225,046)	32,254.8 (2.41–129,869)	0.662
Tumor size, cm	9.3 (4–20)	8.3 (3–21.5)	0.713
Tumor number^a^			0.127
Single	16 (76.2%)	21 (87.5%)	
Multiple	5 (23.8%)	3 (12.5%)	
Vascular invasion^a^	12 (57.1%)	15 (62.5%)	0.714
Organ invasion^a^	7 (33.3%)	5 (20.8%)	0.344
Liver cirrhosis^a^			0.705
Yes	3 (14.3%)	5 (20.8%)	
No	18 (85.7%)	19 (79.2%)	

Values in parentheses are medians with range unless indicated otherwise

*HBV* hepatitis B virus, *TACE* transhepatic arterial chemotherapy and embolization, *AST* aspartate aminotransferase, *ALT* alanine transaminase, *AFP* alpha fetal protein

^a^Values are number with percentages

**Table 3:** Comparison of perioperative and long-term outcomes between DAW and DW group

	DAW group	DW group	*P* value
	*N* = 21	*N* = 24	
Perioperative mortality^a^	0	0	N.A.
Postoperative complication^a^	6 (28.6%)	5 (20.8%)	0.398
Recurrence^a^			
Intrahepatic	10 (47.6%)	15 (62.5%)	0.316
Peritoneal dissemination	1 (4.8%)	7 (29.2%)	0.037
Lung	6 (28.6%)	4 (16.7%)	0.274
Bone	2 (9.5%)	1 (4.2%)	0.449
Brain	0	0	N.A.

Values in parentheses are medians with range unless indicated otherwise

*N.A.* not applicable

^a^Values are number with percentages

Follow-up of patients ranged between 1.5 and 125.5 months. The OS rate at 1, 3, and 5 years was 71.4, 33.3, and 23.8%, respectively, among patients in the DAW group, compared to 50.0, 25.0, and 12.5%, respectively, among patients in the DW group. The median OS was 24.0 months for the DAW group and 11.6 months for the DW (*P* = 0.062). The DFS rate at 1, 3, and 5 years was 47.6, 23.8, and 14.3%, respectively, among patients in the DAW group, and 29.2, 12.5, and 8.3%, respectively, among patients in the DW group. The median DFS was 11.5 months in the DAW group, compared to 4.6 months in the DW group (*P* = 0.006). The recurrence rate of HCC was 76.2% in the DAW group and 62.5% in the DW group (*P* = 0.322). The pattern of recurrence was more likely to be extrahepatic or concomitant intrahepatic and extrahepatic, including metastases to the lung, peritoneal cavity, bone, and brain (Table 3). The incidence of peritoneal dissemination was significantly higher in the DW than in the DAW group (*P* = 0.037). The intrahepatic recurrence rate was 47.6% in the DAW group and 62.5% in the DW group (*P* = 0.483).

**Fig. 5:**
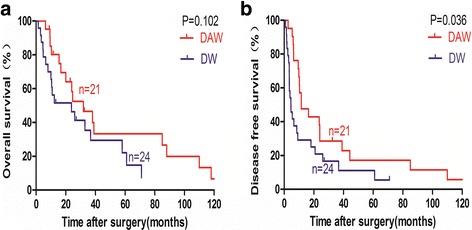
Kaplan–Meier survival curve of OS (**a**) and DFS (**b**) of patients between DAW and DW group. Data for DAW group (*n* = 21) are shown by thick red lines, and data for DW group (*n* = 24) are shown by thick blue line

Using the Kaplan–Meier test, type of treatment and other clinicopathologic parameters, such as age, sex, serum AFP level, serum AST level, serum ALT level, Child-Pugh classification, tumor number, tumor size, hepatitis history, tumor differentiation, organ invasion, vascular invasion, and pre-operation TACE were not identified as being associated with OS (Fig. 5a). The type of treatment, however, was significantly associated with DFS (*P* = 0.036; Fig. 5b). Univariate Cox regression analyses identified better clinical outcomes for patients treated with dehydrated ethanol, although dehydrated ethanol treatment was not identified as an independent predictor of clinical outcome (Table 4).

**Table 4:** Univariate and multivariate analyses of factors associated with OS and DFS

Univariate analysis				
	Overall survival		Disease-free survival	
Variable	Hazard ratio (95% CI)	*P* value	Hazard ratio(95% CI)	*P* value
Age (≤ 47.0 vs. > 47.0)	1.045 (0.494–2.210)	0.908	0.902 (0.442–1.840)	0.777
Gender (female vs. male)	1.618 (0.549–4.767)	0.382	1.076 (0.375–3.089)	0.892
HBsAg (negative vs. positive)	0.756 (0.281–2.029)	0.578	1.254 (0.481–3.268)	0.643
AFP, IU/mL (≤ 400 vs. > 400)	1.000 (0.181–4.645)	0.345	1.669 (0.443–6.289)	0.276
AST, IU/L (≤ 40 vs. > 40)	1.005 (0.996–1.014)	0.257	1.004 (0.997–1.011)	0.229
ALT, IU/L (≤ 40 vs. > 40)	1.004 (0.993–1.015)	0.453	1.001 (0.990–1.012)	0.864
Child-Pugh classification(A vs. B and C)	0.458 (0.062–3.402)	0.445	0.646 (0.152–2.732)	0.552
Tumor size, cm (≤ 10 vs.> 10)	1.062 (0.446–2.528)	0.892	0.690 (0.298–1.598)	0.386
Tumor number (single vs .multiple)	0.849 (0.318–2.267)	0.744	0.571 (0.218–1.493)	0.253
Liver cirrhosis(no vs. yes)	0.819 (0.326–2.059)	0.671	0.605 (0.267–1.370)	0.228
Organ invasion(no vs. yes)	1.158 (0.511–2.625)	0.726	0.912 (0.508–2.298)	0.841
Vascular invasion (no vs. yes)	1.339 (0.535–3.355)	0.533	1.074 (0.504–2.288)	0.854
Pre-operation TACE (no vs. yes)	1.228 (0.415–3.633)	0.711	0.722 (0.251–2.073)	0.544
Type of treatment (DAW vs.DW)	1.828 (0.877–3.810)	0.107	1.951 (1.030–3.693)	0.040
Multivariate analyses				
Type of treatment	N.A.	0.206	N.A.	0.331

Multivariate analysis and Cox proportional hazards regression model were used. Variables were adopted for their prognostic significance by univariate analysis (*P* < 0.05)

*CI* confidence interval, *N.A.* not applicable

## Discussion

A ruptured HCC is considered to be a T4 tumor, according to the TNM staging system, and is associated with a poor clinical prognosis, with tumor cell seeding in the peritoneum increasing the incidence of HCC recurrence [14, 15]. Distilled water lavage during surgery is an established technique to minimize tumor cell seeding after hepatectomy and has been shown to produce positive outcomes [16, 17]. However, cancer cells can survive the mild hypotonicity of the lavage with distilled water [18]; with the effectiveness of the lavage being further limited by contamination of the water in vivo [9] and the “inoculum size” of exfoliated or soiled cancer cells [19]. Dehydrated ethanol injection is a widely used technique for the treatment of nodular-type HCC, with demonstrated safety and feasibility. Ethanol induces cellular dehydration, protein denaturation, and chemical occlusion of small tumor vessels, which causes necrosis of HCC lesions [20]. To our knowledge, our study is the first to propose that the use of dehydrated ethanol in the operative field could provide a similar antitumor role as ethanol injected into the liver for treatment of nodular HCC.

We first investigated the relationship between the duration of ethanol immersion and the depth of liver necrosis. In tissue samples of rat liver, the area of necrosis and coagulative degeneration was visible, with a clear boundary. The depth of necrosis was markedly increased within 10 min, with a maximum depth of 0.2 mm at the time point of 30 min. The depth of degeneration also markedly increased within 5 min to a maximum depth of 1 mm. These results are quite different from studies of in vivo ethanol injection into the liver of animals which have reported a large area of necrosis of 1.5 mm, with a spread of ethanol into an area of up to 29 mm, 15 min after injection of 0.3 ml of ethanol [21, 22]. Reasons for these differences include (1) the increase in the diffusion range of anhydrous alcohol by the pressure of the local injection, and (2) blockage of further absorption of the alcohol by the necrotic layer. Therefore, ethanol immersion does not induce severe necrosis and, thereby lowering the risk for severe complication, such as bleeding, infection, and leakage.

Secondly, we evaluated the safety of our soaking method using a rat model. Due to the difficulty in creating an operative cavity close to the cut surface for soaking within the small surgical field of the rat liver, we covered the cut surface with gauze and applied a continuous infusion of alcohol to the gauze to ensure a sufficient volume of application of alcohol to the cut surface. Soaking was applied for 3 or 5 min, with the diffusion depth of alcohol increasing quickly up to 5 min. The 3 and 5 min durations of soaking were selected to limit operative time, which is essential for survival. In our study, all rats survived the procedure without complication. Blood samples confirmed the safety of our ethanol soaking method.

The safety of the procedure was further confirmed by the very low concentration of serum alcohol identified in patients with HCC rupture who underwent ESFI, with no serum alcohol detected in some patients. Reported serum alcohol concentration after alcohol injection into the liver for treatment of nodular HCC was higher than our levels. The lower level of alcohol absorption in our procedure likely reflects the buffering effect of the layer of necrosis. Moreover, our procedure did not produce additional complications or adverse effects, compared to patients who underwent traditional distilled water lavage. Therefore, our method of dehydrated ethanol soaking can be safely used after curative hepatectomy in patients with ruptured HCC.

OS was comparable among patients in the DAW and DW groups, with our rates being comparable to previously reported values. Taku et al. reported 1-, 3-, and 5-year OS rates, after hepatic resection, of 76.0, 48.6, and 33.9%, respectively [6], with Yang et al. reporting rates of 66.2, 25.1, and 16.8%, respectively [3]. However, in our study, ESFI was predictive of a better DFS, with a mean DFS of 11.5 months for the DAW group compared to 4.6 months for the DW group. Yang et al. reported DFS rates at 1, 3, and 5 years 40.5, 25.8, and 14.8%, respectively. Jing li et al. reported a median OS of 12 months and DFS of 4 months. On univariate analysis, type of treatment was a prognostic factor only for DFS, with no significant prognostic factor identified for OS. It is important to note that ethanol soaking was not retained as a significant prognostic factor on multivariate analysis. Other known prognostic factors, including the Child-Pugh score, tumor size, and satellite nodule in HCC, were not identified as significant factors, suggesting that ruptured HCC may differ from non-ruptured HCC in terms of biological behavior and pathological characteristics. Differences in liver function and extent of cirrhosis between different study cohorts of patients with ruptured HCC would influence, to some degree, prognostic factors identified on univariate and multivariate analyses. This phenomenon is clearly evident when reviewing previously reported prognostic factors. Shogo et al. reported that the Child-Pugh B was the only independent risk factor of unfavorable OS after hepatic resection for ruptured HCC [23]. A multivariate analysis performed by Ou et al. identified tumor size, and the number of tumor modules was the only independent factor influencing long-term postoperative survival among patients with ruptured HCC [15]. Jing Li et al. investigated nine variables selected on multivariate analysis to determine the prognostic predictors of survival in patients with spontaneously ruptured HCC, with only radical resection retained as a prognostic factor of OS and DFS [4].

Finally, several limitations of our study warrant mention. Foremost, the clinical data in our study was retrospectively analyzed, and the number of patients with ruptured HCC was relatively small. In addition, although DFS was significantly different between DAW and DW groups, the treatment type failed to be a prognostic factor of postoperative survival.

## Conclusion

In summary, for the first time, we presented a novel and safe method to lower the risk of seeding of metastatic cells after liver resection for ruptured HCC and to improve the therapeutic effects of hepatic resection. This method could potentially be used not only for spontaneous rupture of HCC but also for intraoperative iatrogenic rupture of HCC and even in cases in which tumor cell shedding in a local area is suspected. Further studies are needed to evaluate the effect of these methods in hepatic resection for the ruptured HCC patients.

## Additional file

Additional file 1: **Table S1.** Blood ethanol concentration of patients after dehydrated ethanol soaked on cut surface of liver during partial hepatectomy. (DOCX 15 kb)

## Abbreviations

AFP: Alpha fetal protein
ALB: Albumin
ALT: Alanine transaminase
AST: Aspartate aminotransferase
HBV: Hepatitis B virus
HCC: Hepatocellular carcinoma
TACE: Transhepatic arterial chemotherapy and embolization
TB: Total bilirubin
