# Supplementary material for: Safety and efficacy of dehydrated ethanol soaking of the operative field in the treatment of spontaneous hepatocellular carcinoma rupture
Source: World J Surg Oncol. 2018 Apr 26;16:86. doi: 10.1186/s12957-018-1390-x (PMC5918920; doi:10.1186/s12957-018-1390-x)
Supplement: Supplementary file 1 — Table S1. Blood ethanol concentration of patients after dehydrated ethanol soaked on cut surface of liver during partial hepatectomy. (DOCX 15 kb) [file 12957_2018_1390_MOESM1_ESM.docx]

| **Supplemental table** Blood ethanol concentration of patients after dehydrated ethanol soaked on cut surface of liver during partial hepatectomy | | | | | | |
| --- | --- | --- | --- | --- | --- | --- |
| Time (min) | Blood ethanol concentration(mg/dL) | | | | | |
|  | Patient 1 | Patient 2 | Patient 3 | Patient 4 | Patient 5 | Patient 6 |
| 0 | undetectable | undetectable | undetectable | undetectable | undetectable | undetectable |
| 5 | undetectable | 19.6 | 21.6 | 25.6 | 30.1 | 18.9 |
| 15 | 26 | 8.0 | 12.5 | 16.5 | 19.6 | 12.5 |
| 60 | 4.3 | undetectable | 8.6 | 10.1 | 6.9 | undetectable |
| 120 | 2.3 | undetectable | undetectable | undetectable | 2.6 | undetectable |
| The minimum detectable concentration is 0.06mg/ dL | | | | | |  |
